# An Unusual Presentation of Primary Hyperparathyroidism: Pathological Fracture

**DOI:** 10.1155/2011/521578

**Published:** 2012-01-26

**Authors:** Ben Abdelghani Khaoula, Ben Abdelghani Kaouther, Chelly Ines, Turki Sami, Leith Zakraoui, Adel Khedher

**Affiliations:** ^1^Department of Internal Medicine, Charles Nicolle Hospital, University of Tunis El Manar, Tunis 1068, Tunisia; ^2^Department of Rheumatology, Mongi Slim Hospital, University of Tunis El Manar, Sidi Daoued, La Marsa 2034, Tunisia; ^3^Department of Anatomopathology, La Rabta Hospital, University of Tunis El Manar, Tunis 1068, Tunisia

## Abstract

Primary hyperparathyroidism revealed by a pathological fracture is very uncommon. We present a case of a 54-year-old female patient who was admitted with fracture of her right femur. She underwent closed intramedullary reconstruction nailing with bipolar locking. The pathological findings confirmed the diagnosis of primary hyperparathyroidism with brown tumor. Further tests showed increased both calcium level and PTH level. A parathyroidectomy was performed. She made an uneventful recovery and was discharged to home.

## 1. Introduction

Nowadays, the occurrence of brown tumor lesions caused by long-lasting primary hyperparathyroidism (HPTP) is very rare, since measuring serum calcium became available routinely in the mid 1970s. It is a tumor-like lesion, often presenting with bone pain or by pathological fracture. Conversely, a pathological fracture revealing a PHPT is not commonly described. Presented here is an interesting case of PHPT revealed by a pathological fracture.

## 2. Observation

**Figure 1:**
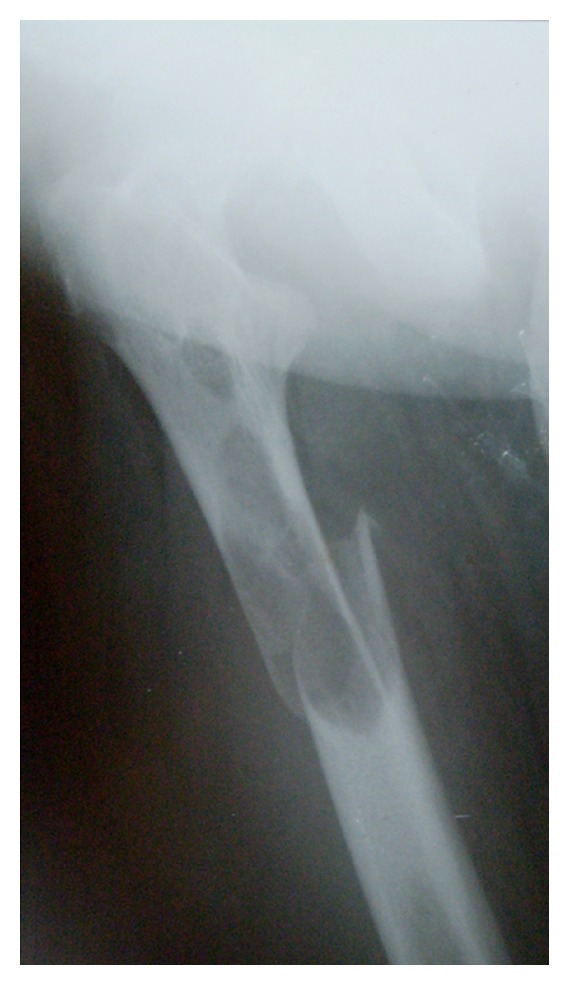
Radiographs of pathological subtrochanteric fracture of right femur.

**Figure 2:**
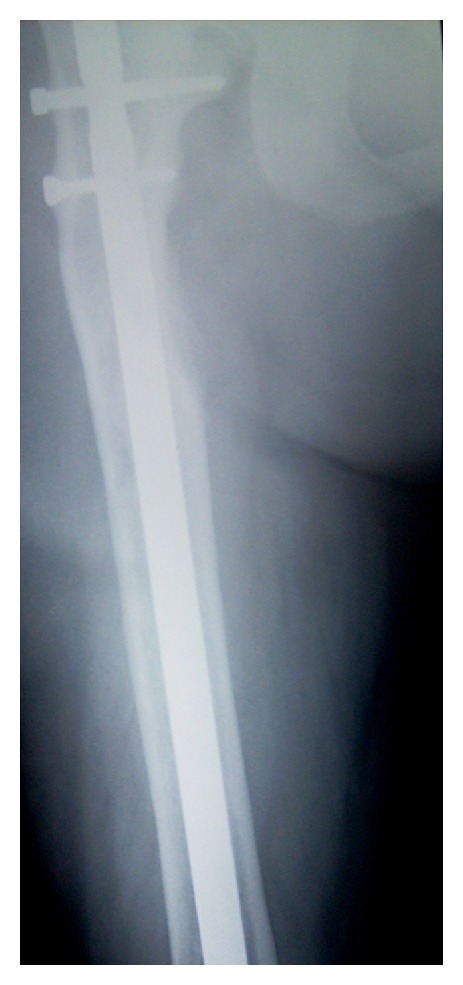
Reconstruction intramedullary nail right femur.

**Figure 3:**
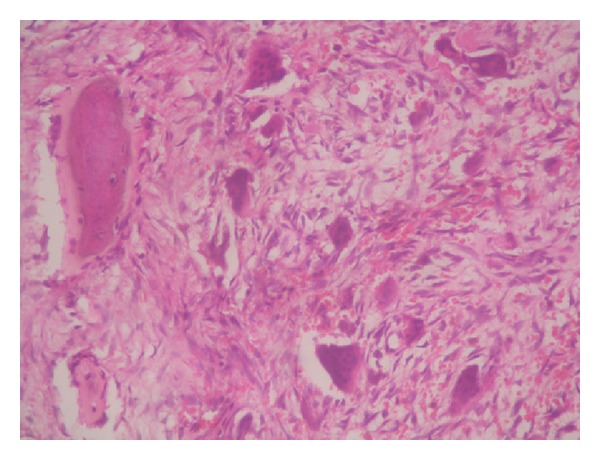
Histopathological examination showing giant cells mixed with mononuclear cells devoid of atypia consisting with hyperparathyroidism (high magnification).

**Figure 4:**
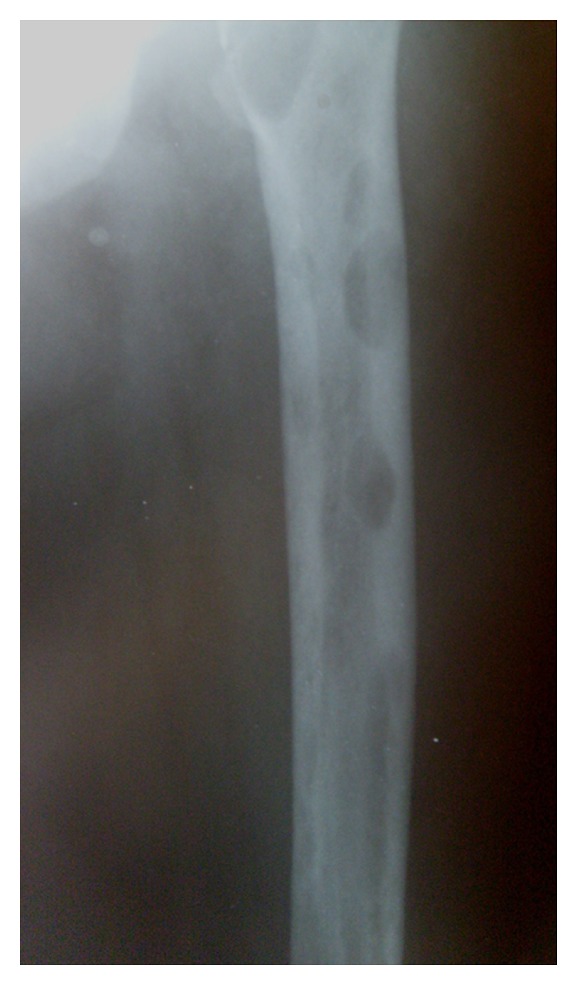
Radiograph of the left femur showing multiple lytic lesions.

A 54-year-old lady presented to the orthopaedic department after tripping on a step and falling onto her right side. She was otherwise fit and well. Radiographs confirmed a subtrochanteric fracture of her femur through an area of lytic bone (Figure 1). Surgical intervention was conducted for the pathologic fracture: the patient underwent closed intramedullary reconstruction nailing of her femur with bipolar static locking (Figure 2). She made an uncomplicated recovery from surgery and the histological findings confirmed the diagnosis of hyperparathyroidism with brown tumor (Figure 3). Additional radiographs showed asymptomatic lytic lesions in the left femur (Figure 4). Plain radiographies of the hands, skull, clavicle, and sacroiliac joints were performed but no abnormalities were found. The patient bone profile revealed markedly raised calcium of 3.3 mmol/l (corrected range: 2.15–2.60). The creatinine level was within normal limits. A PTH assay objectified a circulating level of PTH of 830 pg/mL (13–54) and the diagnosis of PHPT was then confirmed. A cervical ultrasound had identified an adenoma in the left inferior parathyroid gland. A parathyroidectomy was performed and a large (13 g) parathyroid was removed and confirmed to be a benign parathyroid adenoma on histological examination. Despite a transient postoperative hypocalcaemia, the patient made an uneventful recovery and was discharged to home. Two weeks later, the level of PTH was within normal (48 pg/mL).

## 3. Discussion

The incidence of PHPT is quoted at 2–10/10 000 of the population. Of these, 50–70% are now routinely detected on incidental biochemical assays. Up to one third of patients may be asymptomatic. Bone pain and tenderness are seen in PHPT but occur more commonly in secondary hyperparathyroidism [1]. Very rarely, fracture occurs in HPT. The crude fracture rate in patients known to have PHPT has been documented at 15/1000 person years compared to 8/1000 in controls [2]. However, PHPT revealed by pathological fracture, as in our case, is very uncommon [1–4]. Our patient had a history of falling, and considering her age, osteoporosis was probably associated to some extent. Interestingly, no correlation had been identified between fracture risk and preoperative calcium levels or the weight of the diseased parathyroid removed at surgery [2]. These fractures are due to brown tumor which is a rare complication of PHPT. Brown tumors are benign focal bone lesions caused by increased osteoclastic activity and fibroblastic proliferation, encountered in primary or more rarely secondary hyperparathyroidism [5]. These tumors may appear in any bone but are frequently found in the facial bones and jaws, sternum, pelvis, ribs, femur, and rarely the vertebrae [6]. The histological findings of the tumor are similar to those of giant cell tumors and aneurismal bone cyst and may cause confusion in diagnosis [7]. Nevertheless, because of the difficulty of microscopic differential diagnosis, the clinical presentation and the biochemical findings should be considered carefully for making the correct diagnosis. The increased PTH value is the determinant in diagnosis. In our patient, the diagnosis was established according to the histological findings. In X-rays, brown tumors are seen as lytic lesions with regular borders. The cortex can be seen as narrowed and extended; however, there is no penetration. These large bone defects increase the spontaneous fracture risk. Our patient's radiologic views were also consistent with these findings. CT is useful for evaluating brown tumors of lower limbs to show their uniform tissue density and the contrast enhancement of the lesion. However, when CT shows a fluid-filled cyst, local surgery must be performed to prevent a pathological fracture. Brown tumors are hypervascular and therefore enhance on MRI, and intensely active in bone scintigraphy [8]. In addition to the surgical stabilization of the femur, aggressive treatment of hyperparathyroidism is also necessary to achieve successful outcomes. Thus, the increased risk of fracture disappears within a year following surgery suggesting a quick restoration of bone biomechanical competence [7].

## 4. Conclusion

Brown tumor is a benign clinical entity appearing as a skeletal manifestation of hyperparathyroidism. It is an uncommon cause of pathological fractures. Stabilization of the fracture and aggressive treatment of hyperparathyroidism are the key points of the treatment of this rare clinical entity. We believe that the present case will contribute to available knowledge for the differential diagnosis of pathological fractures.
